# Association between intrinsic capacity and chronic lung disease among middle-aged and older adults: evidence from CHARLS and ELSA

**DOI:** 10.1186/s12889-026-27756-9

**Published:** 2026-05-21

**Authors:** Qing-Ao Xiao, Bo-Tian Song, Wei Hong, Yu-Long Zhao, Zhang Cheng, Jia-Chen Zhang, Liangrong Shi

**Affiliations:** Department of Radiology, Xiangya Hospital, Central South University, Changsha, China

**Keywords:** Chronic Lung Disease, Intrinsic Capacity, CHARLS, ELSA

## Abstract

**Background:**

Impaired Intrinsic capacity (IC) has been closely associated with the occurrence of various diseases. However, the relationship between impaired IC and chronic lung disease (CLD) remains unclear. This study aims to investigate the association between impaired IC and CLD among individuals aged 50 and above, as well as the potential country-specific differences.

**Methods:**

This study utilized longitudinal follow-up data from the China Health and Retirement Longitudinal Study (CHARLS, 2011–2018) and the English Longitudinal Study of Ageing (ELSA, 2012–2018). Cox proportional hazards regression analyses were subsequently conducted to evaluate the association between IC impaired and risk of CLD. Subgroup analyses were performed to identify potential effect modifiers. The Restricted cubic splines (RCS)analysis was conducted to clarify whether there is a linear relationship between IC scores and CLD.

**Results:**

A total of 7,897 participants were included, comprising 3,540 individuals from CHARLS and 4,357 from ELSA. The incidence of CLD was 10.6% in CHARLS and 3.1% in ELSA. After adjusting for covariates, participants with impaired IC exhibited a significantly higher risk of developing CLD compared to those with normal IC, with hazard ratio (HR) of 1.738 (95% confidence interval [CI], 1.371–2.205) in CHARLS and 1.983 (95% CI, 1.352–2.908) in ELSA. The results of the RCS analysis indicate that IC scores are negatively correlated with CLD (*P* for nonlinear > 0.05). Stratified analyses indicated that gender significantly modified this association in CHARLS (*P* for interaction < 0.05), whereas no such effect modification was observed in ELSA. Sensitivity analyses yielded results consistent with the primary analyses.

**Conclusion:**

Impaired IC among middle-aged and older adults is closely associated with the incidence of CLD in both CHARLS and ELSA. In the Chinese population, women exhibit a higher risk of developing CLD following impaired IC compared to men.

**Supplementary Information:**

The online version contains supplementary material available at 10.1186/s12889-026-27756-9.

## Background

Chronic lung disease (CLD) poses formidable challenge for middle-aged and older adults. CLD encompasses chronic bronchitis, emphysema, or pulmonale, asthma, and other related conditions, with the risk of onset increasing markedly with age [1]. Globally, by 2021, an estimated 468 million people were affected by CLD [2]. In China, a study showed that approximately 100 million individuals suffer from chronic obstructive pulmonary disease (COPD), making it the third most common chronic disease after hypertension and diabetes [1]. The occurrence of CLD is often accompanied by depression [3] and impairments in activities of daily living [4], imposing substantial burdens on individuals and society. Hence, assessing the risk of CLD development in middle-aged and older populations is crucial for prevention efforts.

Intrinsic Capacity (IC) is a concept proposed by the World Health Organization (WHO) in the 2015 Global Report on Aging and Health [5]. IC represents the core of older individual’s functional abilities and can be subdivided into five domains: locomotion, vitality, psychological status, cognition, and sensory [6]. Previous studies have demonstrated that IC is closely associated with the incidence of cardiovascular diseases [7], sarcopenia [8], and falls [9]. Therefore, early assessment and timely intervention targeting IC have the potential to significantly delay functional decline, improve quality of life among the elderly, and promote healthy aging.

Previous study has indicated that components of IC, such as hearing and vision impairments, are associated with an increased risk of various chronic diseases in the Chinese population, with the risk of developing CLD heightened by approximately 53% [10]. This research attributes the increased risk of chronic diseases associated with vision and hearing impairments to factors such as systemic chronic inflammation, abnormal immune activation, and vascular dysregulation [10]. Some studies conducted on non-Chinese populations have also demonstrated a close association between CLD and hearing impairment [11], depression [12], and visual impairment [13].

However, some studies have indicated that there are population differences in the associations between CLD and the components of IC. For instance, research on the genetic correlation between depression and CLD has shown a significant genetic association in European populations, while such an association does not exist in East Asian populations [14]. In addition, the associations between the components of IC also exhibit population differences. For instance, depression and body mass index (BMI) show a negative correlation in East Asian populations, whereas a positive correlation is observed in European populations [15, 16]. More importantly, there are complex interrelationships among the components of IC. For example, hearing and visual impairments frequently coexist in older adults [17], and both are associated with cognitive decline [18, 19]. Depression is also linked to both cognitive impairment [20] and abnormal BMI [21]. Therefore, focusing on a single component may underestimate the true risk or fail to capture the combined exposure of multiple impairments. As a composite measure, IC may provide a more comprehensive assessment of the associations between its components and CLD. However, there is currently a lack of evidence in this area. In addition, due to the population differences in certain components of IC, it remains unclear whether the association between IC impairment and CLD varies across different populations.

In this study, we utilized data from the China Health and Retirement Longitudinal Study (CHARLS) and the English Longitudinal Study of Ageing (ELSA) to investigate the relationship between IC impairments and the risk of CLD. We aim to clarify the association between IC impairment and the occurrence of CLD among individuals aged 50 and above, as well as to identify the differences between China and UK populations.

## Materials and methods

### Participants

**Fig. 1:**
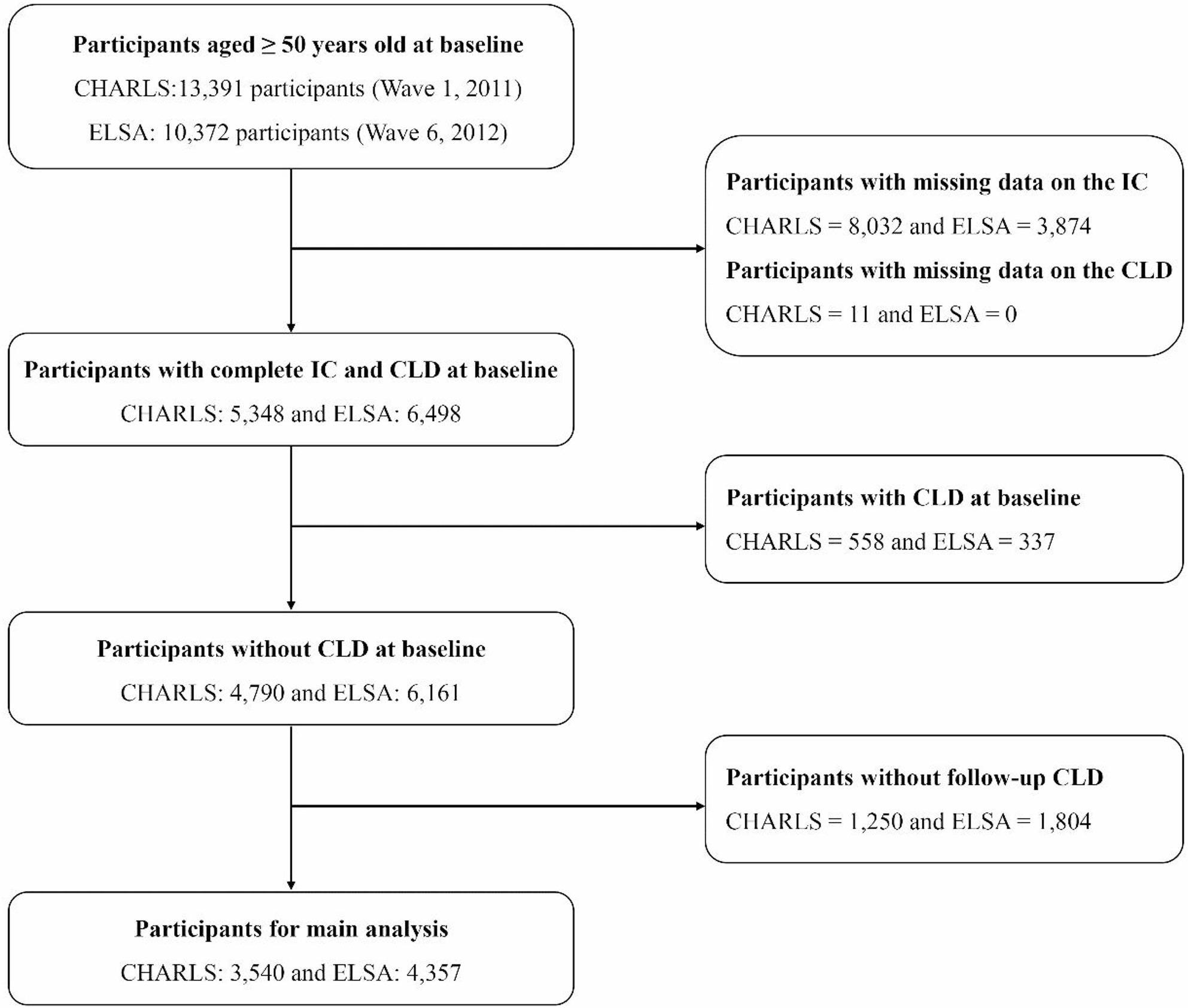
Flowchart of this study. Abbreviations: CHARLS, China Health and Retirement Longitudinal Study; ELSA, English Longitudinal Study of Ageing; CLD, Chronic Lung Disease; IC, Intrinsic Capacity

The CHARLS and ELSA are nationally representative cohort studies from China and the United Kingdom, respectively, containing comprehensive information on the health, economic status, and other characteristics of middle-aged and older adults in these countries [22, 23]. The original studies obtained written informed consent from participants at the time of data collection and adhered to the standards set forth in the Declaration of Helsinki. The CHARLS and the ELSA were approved for public release by the Biomedical Ethics Review Committee of Peking University and the London Multicenter Research Ethics Committee, respectively. In the present study, we utilized the publicly available datasets, and since no individual patient identifiable information was involved in the analysis, no additional ethical review was required. In this study, the data from the 2011 to 2018 waves of CHARLS (wave 1 to wave 4) and the 2012 to 2018 waves of ELSA (wave 6 to wave 9) were utilized. The flowchart of the research is shown in Fig. 1.

### Assessment of chronic lung disease

In CHARLS, participants were asked the question, “Have you ever been diagnosed by a doctor with CLD such as chronic bronchitis or emphysema (excluding tumors or cancer)?” Similarly, in ELSA, respondents were asked whether they had chronic bronchitis, emphysema, or COPD. Participants who answered “Yes” to these questions were classified as having CLD, otherwise, classified as non-CLD.

### Assessment of intrinsic capacity

IC was constructed based on five domains: locomotion, vitality, sensory, cognition, and psychology. Based on previous studies, we divided sensory function into two components: hearing and vision [24]. Each domain was scored dichotomously as 0 or 1, where 1 indicated normal function and 0 indicated declined function, resulting in a total score ranging from 0 to 6 [25]. In this study, individuals with a total IC score of five or less were defined as having impaired IC, while those with an IC score of six were classified as normal [25]. The IC assessment results of participants from the CHARLS study in 2011 and the ELSA study in 2012 were used as the baseline for this research. The specific scoring criteria were as follows:

1. Locomotion: Participants independently completed the five-times sit-to-stand test, and the corresponding completion time was recorded. In CHARLS, a time ≤14 seconds was assigned 1 point, while a time >14 seconds was scored 0[9]. For ELSA, based on previous literature, a cutoff of ≤16.7 seconds was assigned 1 point, and >16.7 seconds was scored 0 [23].
2. Vitality: BMI was calculated for each participant. BMI ≤18.5 kg/m² was scored 0, indicating impaired vitality, whereas a BMI >18.5 kg/m² was scored 1[25].
3. Hearing: Self-reported hearing ability was classified into five levels in both CHARLS and ELSA: excellent, very good, good, fair, and poor. Participants reporting "poor" hearing were assigned 0 points; all other responses were assigned 1 point [26].
4. Vision: Participants were asked to report their near and distance vision. In CHARLS, individuals reporting "poor" vision were assigned 0 points, and others 1 point [26]. In wave 6 of ELSA, an additional category of "blind" was available; therefore, participants reporting "poor" or "blind" vision were assigned 0 points, while others were assigned 1 point [23].
5. Cognition: Cognitive function was assessed through memory and mental status. For memory evaluation, both ELSA and CHARLS participants were randomly given a list of 10 words to recall immediately (immediate recall, scored 0-10) and after a 10-minute delay (delayed recall, scored 0-10). The mean of immediate and delayed recall scores was calculated as the memory score [25]. Mental status in CHARLS included tests such as serial subtraction of 7 from 100 five consecutive times, awareness of current month, date, year, day of the week, season, and a drawing test, with a total score ranging from 0 to 11. In wave 6 of ELSA, tests for serial subtraction, season identification, and drawing were unavailable; therefore, consistent with previous ELSA study, mental status was assessed solely based on participants’ correct identification of the current month, date, year, and day of the week, scored from 0 to 4 [23]. The sum of memory and mental status scores was then calculated for each participant in both cohorts. Using the mean minus one standard deviation as a cutoff, participants scoring above this threshold were considered cognitively normal (scored 1), whereas those scoring at or below the cutoff were defined as cognitively impaired (scored 0) [25].
6. Psychology: Depression was assessed using the Center for Epidemiologic Studies Depression Scale (CESD), with CESD-10 applied in CHARLS and CESD-8 in ELSA, both scales have been widely used in studies of depression within these cohorts [27–29]. The CESD-10 total score ranges from 0 to 30; in CHARLS, participants scoring ≥10 were assigned 0 points (indicating depressive symptoms), while those scoring <10 were assigned 1 point [29]. The CESD-8 total score ranges from 0 to 8; in ELSA, participants scoring ≥3 were assigned 0 points, and those scoring <3 were assigned 1 point [30].

### Covariates

In this study, we included several confounding variables based on previous literature [25, 27, 31], comprising (1) demographic characteristics: gender, age, marital status, and educational level; (2) lifestyle factors: smoking, alcohol consumption, sleep duration, and physical activity; and (3) health status: hypertension, diabetes, heart disease, and BMI. Detailed descriptions and categorizations of these covariates are provided in the Supplementary methods. To minimize bias, participants with missing covariate data were not excluded in the initial analysis. The number and proportion of missing values for each variable are presented in Table S1.

### Statistical analysis

Continuous variables were presented as mean ± standard deviation or median with interquartile range, depending on whether they conformed to a normal distribution, while categorical variables were expressed as frequencies and percentages. Cox proportional hazards models were employed to estimate hazard ratio (HR) and 95% confidence interval (CI) for the association between impaired IC and risk of CLD. In this study, four models were constructed: Model 1 was unadjusted; Model 2 adjusted for demographic characteristics; Model 3 further adjusted for lifestyle factors; and Model 4 additionally adjusted for health status. Kaplan-Meier curves were generated to illustrate the cumulative incidence of CLD across different IC statuses, with differences compared using the log-rank test. The proportional hazards (PH) assumption of the Cox models was assessed using Schoenfeld residuals, and *p*-value greater than 0.05 indicated no violation of the PH assumption. Secondly, we performed restricted cubic splines (RCS) to determine whether the association between IC scores and the risk of CLD is linear. Subgroup analyses were performed to assess whether the effect of IC on CLD risk was modified by other variables. Based on previous studies, we selected covariates including age, gender, educational level, physical activity, smoking status, alcohol consumption, sleep duration, hypertension, and diabetes to conduct subgroup analyses [27].

To ensure the robustness of the results, sensitivity analyses were conducted. Multiple imputation by chained equations (MICE) was applied to impute missing data 20 times, followed by repeated statistical analyses to address potential selection bias caused by missing data.

## Results

### The characteristics of study participants

A total of 7,897 participants were included in this study, of whom 3,540 were from CHARLS and 4,357 from ELSA. In the CHARLS data, the IC normal group included 1,245 individuals, with a total follow-up of 8,562 person-years. Among them, 102 cases of CLD were observed, resulting in an incidence rate of 11.9 per 1,000 person-years. The IC impaired group comprised 2,295 individuals, with a cumulative follow-up of 15,578 person-years, resulting in 345 cases of CLD and an incidence rate of 22.1 per 1,000 person-years. When using the IC normal group as a reference, the relative risk (RR) of experiencing the outcome event in the IC impaired group was 1.859 (95% CI: 1.491–2.319), indicating that IC impairment is associated with a higher risk of CLD. In the ELSA data, the IC normal group included 3,037 individuals, with a follow-up of 18,124 person-years, during which 68 cases of CLD occurred, leading to an incidence rate of 3.75 per 1,000 person-years. The IC impaired group consisted of 1,320 individuals, with a follow-up of 7,776 person-years, and 66 cases of CLD were reported, resulting in an incidence rate of 8.49 per 1,000 person-years. The RR of the impaired group compared to the normal group was 2.262 (95% CI: 1.612–3.174), further indicating that IC impairment significantly increases the risk of CLD.

Except for diabetes prevalence, which did not differ significantly between the two cohorts, all other variables showed statistically significant differences (*P* < 0.05). Compared with ELSA participants, those in CHARLS were younger (median age 59 vs. 65 years), had shorter sleep duration (median 6.5 vs. 7 hours), and a lower BMI (23.35 vs. 27.35 kg/m^2^). Notably, the IC score was higher in ELSA than in CHARLS (6 vs. 5 scores). Baseline characteristics of the study participants are shown in Table 1. Additionally, we compared the baseline characteristics between the included and excluded participants in this study, as detailed in Table S2. Baseline characteristics stratified by IC groups within each cohort are presented in Table S3.

**Table 1:** The characteristics of study participants

Variables	Total (*n* = 7897)	CHARLS (*n* = 3540)	ELSA (*n* = 4357)	*P* value
Age, Median (IQR), years	62 (57, 68)	59 (55, 65)	65 (59, 71)	< 0.001
Gender, n (%)				< 0.001
Female	4114 (52.096)	1706 (48.192)	2408 (55.267)	
Male	3783 (47.904)	1834 (51.808)	1949 (44.733)	
Marital status, n (%)				< 0.001
Married	4448 (56.332)	3181 (89.859)	1267 (29.086)	
Other	3448 (43.668)	359 (10.141)	3089 (70.914)	
Education status, n (%)				< 0.001
High school and below	5489 (72.462)	3499 (98.842)	1990 (49.318)	
College and above	2086 (27.538)	41 (1.158)	2045 (50.682)	
Smoking status, n (%)				< 0.001
No	3809 (48.234)	2011 (56.808)	1798 (41.267)	
Yes	4088 (51.766)	1529 (43.192)	2559 (58.733)	
Sleeping time, Median (IQR), hours	7 (6, 8)	6.5 (5, 8)	7 (6, 8)	< 0.001
Drinking status, n (%)				< 0.001
No	2669 (35.922)	2296 (68.722)	373 (9.122)	
Yes	4761 (64.078)	1045 (31.278)	3716 (90.878)	
PA, n (%)				< 0.001
Moderate-to-vigorous	3595 (45.524)	2483 (70.141)	1112 (25.522)	
Other	4302 (54.476)	1057 (29.859)	3245 (74.478)	
BMI, Median (IQR), kg/m^2^	25.540 (22.696, 28.826)	23.350 (21.072, 25.907)	27.346 (24.756, 30.599)	< 0.001
Diabetes, n (%)				0.081
No	7275 (92.334)	3273 (92.93)	4002 (91.852)	
Yes	604 (7.666)	249 (7.07)	355 (8.148)	
Hypertension, n (%)				< 0.001
No	5283 (67.018)	2571 (72.915)	2712 (62.245)	
Yes	2600 (32.982)	955 (27.085)	1645 (37.755)	
Heart disease, n (%)				0.002
No	6810 (86.345)	3096 (87.705)	3714 (85.242)	
Yes	1077 (13.655)	434 (12.295)	643 (14.758)	
Cancer, n (%)				< 0.001
No	7456 (94.487)	3507 (99.236)	3949 (90.636)	
Yes	435 (5.513)	27 (0.764)	408 (9.364)	
IC, Median (IQR)	6 (5, 6)	5 (4, 6)	6 (5, 6)	< 0.001
CLD, n (%)				< 0.001
No	7316 (92.643)	3093 (87.373)	4223 (96.924)	
Yes	581 (7.357)	447 (12.627)	134 (3.076)	

Abbreviations: *BMI* Body Mass Index, *CHARLS* China Health and Retirement Longitudinal Study, *CLD* Chronic Lung Disease, *ELSA* English Longitudinal Study of Ageing, *IC* Intrinsic Capacity, *IQR* Interquartile range, *PA* Physical Activity

### Association between impaired IC and CLD

A total of 581 participants developed CLD, including 447 from the CHARLS cohort and 134 from the ELSA cohort. The association between baseline impaired IC and the risk of CLD is presented in Table 2. Results from the Schoenfeld residual tests indicated that the PH assumption was not violated for the key variable IC across all cohorts (Table S4).

**Table 2:** Association of IC impaired with the risk of chronic lung disease

Term	Event/*n*	Model 1		Model 2		Model 3		Model 4	
		HR (95%CI)	*P* value	HR (95%CI)	*P* value	HR (95%CI)	*P* value	HR (95%CI)	*P* value
CHARLS									
IC normal	102/1245	Ref		Ref		Ref		Ref	
IC impaired	345/2295	1.902(1.525–2.373)	< 0.001	1.942(1.552–2.429)	< 0.001	1.825(1.447–2.302)	< 0.001	1.738(1.371–2.205)	< 0.001
ELSA									
IC normal	68/3037	Ref		Ref		Ref		Ref	
IC impaired	66/1320	2.275(1.622–3.192)	< 0.001	2.179(1.533–3.098)	< 0.001	1.953(1.335–2.856)	0.001	1.983(1.352–2.908)	< 0.001

*Abbreviations*: *CHARLS* China Health and Retirement Longitudinal Study, *CI* Confidence Interval, *ELSA* English Longitudinal Study of Ageing, *HR* Hazard Ratio, *IC* Intrinsic Capacity, *Ref* Reference

Model 1: unadjusted

Model 2: adjusted for Age, Sex, Education status and Marital status

Model 3: adjusted for Age, Sex, Education status, Marital status, Smoking status, Drinking status, Sleeping time, and Physical Activity

Model 4: adjusted for Age, Sex, Education status, Marital status, Smoking status, Drinking status, Sleeping time, Physical activity, Hypertension, Diabetes, BMI, Heart disease and Cancer

In the CHARLS cohort, impaired IC was significantly associated with an increased risk of CLD in the unadjusted model (Model 1: HR = 1.902; 95% CI, 1.525–2.373). This association remained robust after adjustment for demographic characteristics, lifestyle factors, and health status (Model 4: HR = 1.738; 95% CI, 1.371–2.205). Similarly, a significant association was observed in the ELSA cohort (Model 1: HR = 2.275; 95% CI,1.622–3.192), and remained significant after adjusting for demographic characteristics, lifestyle factors, and health status (Model 4: HR = 1.983; 95% CI, 1.352–2.908).

**Fig. 2:**
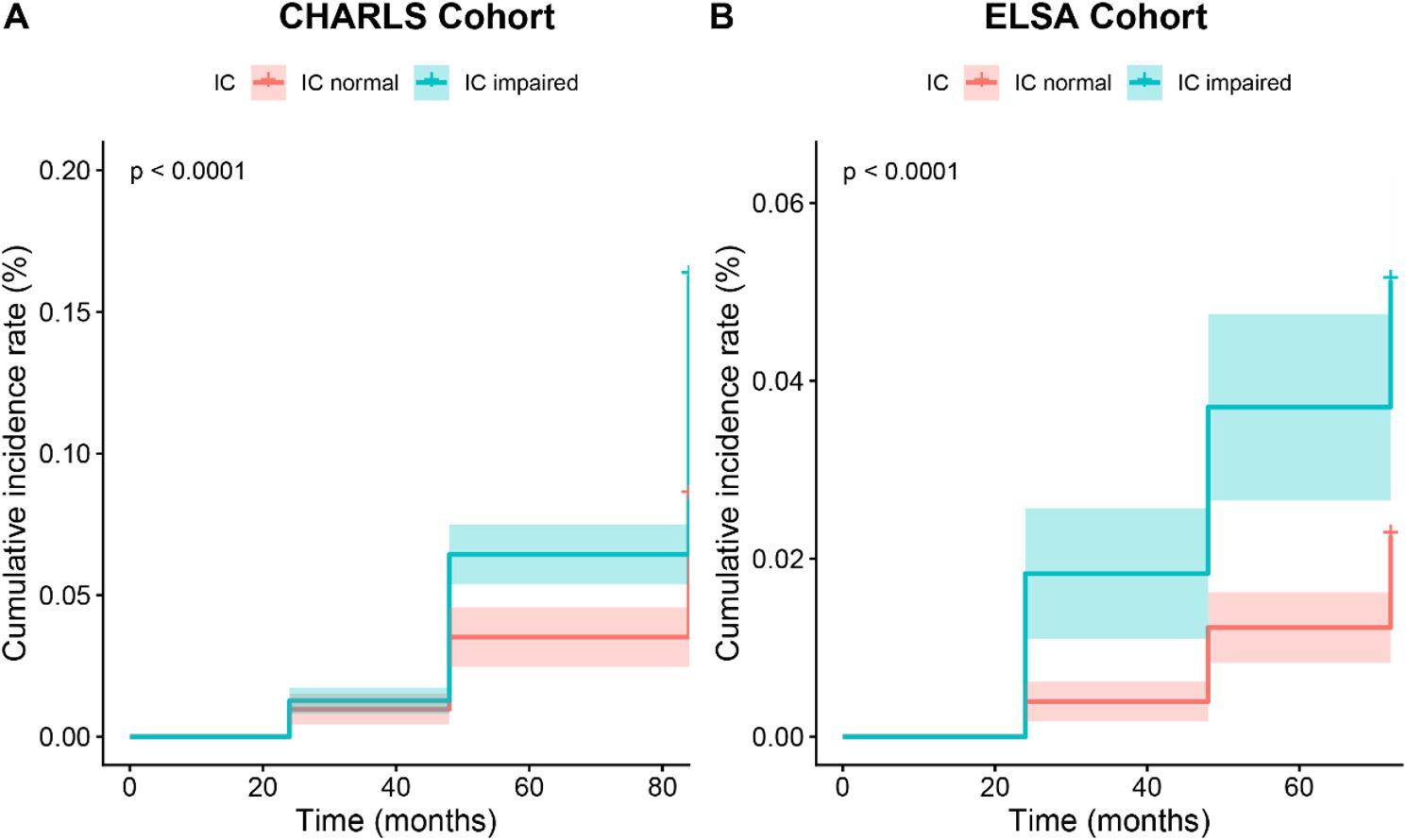
Cumulative risk of chronic lung disease among individuals with different intrinsic capacity. **A** CHARLS cohort; (**B**) ELSA cohort. Abbreviation: CHARLS, China Health and Retirement Longitudinal Study; ELSA, English Longitudinal Study of Ageing; IC, Intrinsic Capacity

**Fig. 3:**
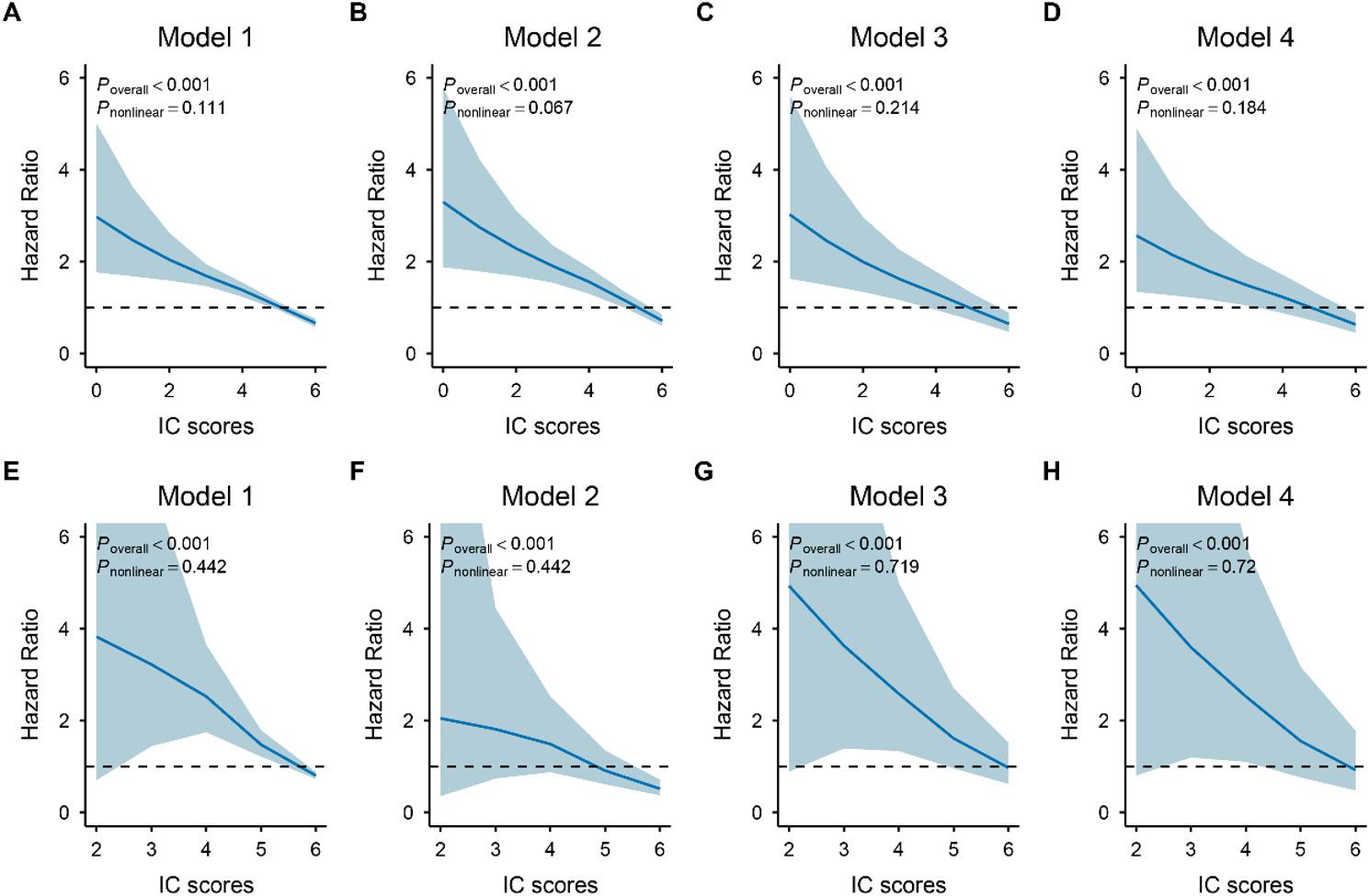
The results of RCS between intrinsic capacity scores and risk of chronic lung disease. **A**-**D**: The results of RCS in CHARLS; **E**-**H**: The results of RCS in ELSA. Model 1: unadjusted. Model 2: adjusted for Age, Gender, Education status and Marital status. Model 3: adjusted for Age, Gender, Education status, Marital status, Smoking status, Drinking status, Sleeping time, and Physical Activity. Model 4: adjusted for Age, Gender, Education status, Marital status, Smoking status, Drinking status, Sleeping time, Physical activity, Hypertension, Diabetes, BMI, Heart disease and Cancer. Abbreviation: CHARLS, China Health and Retirement Longitudinal Study; ELSA, English Longitudinal Study of Ageing; IC, Intrinsic Capacity

These results indicate that, compared with the normal IC group, participants with impaired IC in the CHARLS cohort exhibited approximately a 73.8% increased risk of CLD, whereas those in the ELSA cohort demonstrated a 98.3% higher risk. Cumulative incidence analyses demonstrated that impaired IC was associated with an increased risk of developing CLD in both the CHARLS and ELSA cohorts (Fig. 2). The results of the RCS analysis indicated that as IC scores increase, the risk of developing CLD shows a linear decrease (*P* for nonlinear > 0.05). Furthermore, this linear relationship remains valid even after adjusting for covariates (Fig. 3).

### Subgroup analysis

**Fig. 4:**
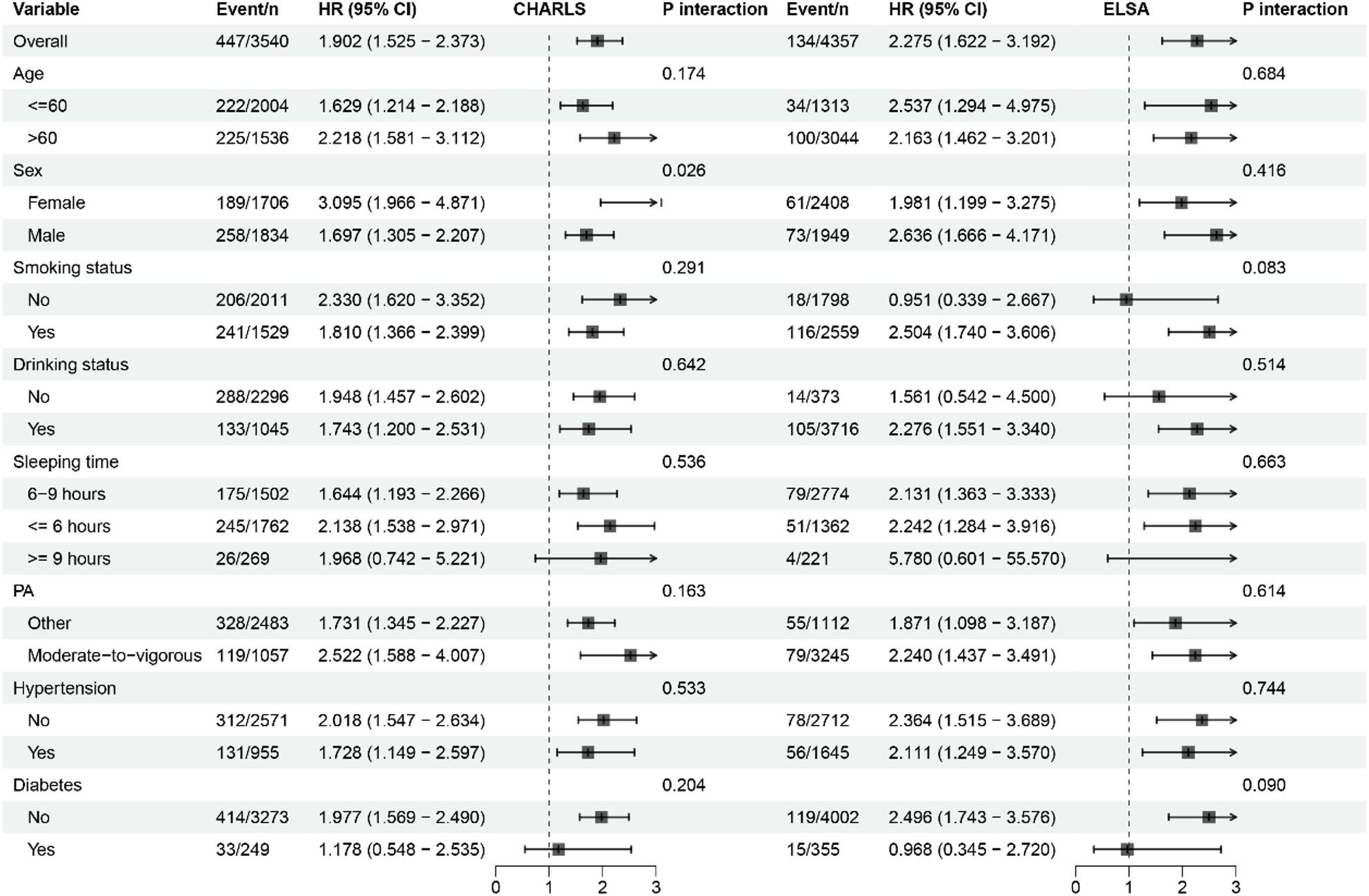
Subgroup analysis of the association between impaired intrinsic capacity and risk of chronic lung disease. Abbreviation: CHARLS, China Health and Retirement Longitudinal Study; ELSA, English Longitudinal Study of Ageing; PA, Physical Activity; IC, Intrinsic Capacity

Stratified analyses were performed to evaluate whether the association between impaired IC and CLD was modified by specific covariates (Fig. 4). A significant effect modification by gender was observed only in the CHARLS cohort (*P* for interaction = 0.026), with females exhibiting a higher risk of developing CLD (HR = 3.095; 95% CI, 1.966–4.871) compared to males among those with impaired IC (HR = 1.697; 95% CI, 1.305–2.207). However, this effect was not observed in the ELSA cohort (Fig. 4). Other covariate subgroups, such as age, smoking status, drinking status, sleeping time, PA, hypertension, and diabetes, did not show significant interaction effects (*P* for interaction > 0.05).

### Sensitivity analysis

Subsequently, sensitivity analyses were conducted, and multiple imputation was further applied to covariates with missing values. The results from the analyses based on multiply imputed data were consistent with the initial findings (Table S5). These results further defined the robustness of the initial analyses.

## Discussion

In this study, we evaluated the association between impaired IC and the risk of CLD incidence using two large national cohort studies. The results demonstrated that middle-aged and older adults with impaired IC had an increased risk of developing CLD.

A cross-sectional study conducted in 2021 among the Chinese population reported significant associations between impaired IC and various diseases, including CLD, kidney disease, stroke, and coronary heart disease [32]. However, that study did not clarify whether impaired IC promotes the onset of CLD. Our study is among the first to demonstrate that impaired IC promotes the development of CLD in middle-aged and older adults. The mechanisms by which impaired IC promotes CLD onset are likely multifactorial. First, previous study has shown that patients with COPD and low BMI have a higher prevalence of COPD compared to those with normal BMI (21.1% vs. 7.5%), along with lower forced expiratory volume in one second (FEV1) levels [33]. Low BMI often indicates malnutrition, which may impair respiratory muscle function and lead to decreased pulmonary function [34]. Additionally, protein deficiency can cause immune dysfunction, increasing susceptibility to pulmonary infections and further damaging lung function [35]. Second, recent research has indicated that early sensory impairments, including vision, hearing, or dual sensory loss, are associated with a higher incidence of CLD [10]. This may be attributable to the chronic inflammatory state often present in individuals with sensory impairments [36]. Chronic inflammation and oxidative stress can promote pulmonary epithelial autophagy and endothelial cell dysfunction, thereby damaging lung tissue and contributing to airway remodeling [37, 38]. Furthermore, depression is one of the factors affecting impaired IC. Numerous studies have shown that patients with depression also have a higher risk of developing CLD, with a bidirectional relationship existing between depression and CLD, often co-occurring in middle-aged and older adults [3, 39]. Evidence from studies involving older adults in the UK suggests that for each one-point increase in the CESD score, the risk of developing CLD rises by approximately 5% [40].

For cognition, studies have shown that CLD increases the risk of cognitive impairment in middle-aged and elderly individuals, which may be attributed to hypoxia caused by the dyspnea associated with COPD [41, 42]. This condition can lead to structural changes in the brain of COPD patients, with significant damage observed in the prefrontal cortex compared to healthy individuals [41]. Notably, it remains unclear whether cognitive impairment itself increases the risk of developing CLD. In assessing locomotion, we employed the five-repetition sit-to-stand test. A study conducted in 2022 demonstrated that this method effectively predicts the five-year mortality rate in individuals with COPD [43]. Relevant research based on Genome-wide association study (GWAS) data indicates a negative correlation between walking speed and the incidence of COPD [44]. This suggests a potential genetic relationship between physical activity and the occurrence of COPD [44]. One possible explanation is that a decrease in physical activity may lead to weakened respiratory muscles and reduced immune function, thereby increasing the likelihood of developing COPD.

Notably, in our study, impaired IC among Chinese females was associated with a higher risk of CLD. However, this phenomenon was not observed in the UK cohort. This discrepancy may be explained by the higher prevalence of depressive symptoms among Chinese women, which is also closely linked to the development of CLD [45]. Additionally, in the middle-aged and elderly population in China, heating and cooking often rely on non-clean fuels such as coal and wood [46]. The incomplete combustion of these fuels produces air pollutants, which may contribute to an increased risk of CLD in women [46]. Further research is warranted to confirm this observation.

This study has several limitations. First, the original cohort studies did not include questionnaires specifically designed to assess individual IC, so the methods used herein may not fully capture the true IC levels of participants. Second, the assessment of CLD relied on self-reported data, which may be subject to recall bias. Finally, the covariates selected in this study were limited, which may have resulted in unmeasured confounding factors influencing the results, such as ambient air pollution, climate change and history of severe respiratory infections. Future research is warranted to further clarify the impact of additional covariates on the outcomes.

## Conclusion

Impaired IC increases the risk of CLD among middle-aged and older adults in both China and the UK. Assessing and maintaining IC may reduce the incidence of CLD. Particular attention should be given to IC in Chinese women, as this group appears more susceptible to CLD development following impaired IC.

## Supplementary Information

Supplementary Material 1.

## Data Availability

CHARLS ( https://charls.pku.edu.cn/ ) and ELSA ( https://www.elsa-project.ac.uk/accessing-elsa-data ) can all be obtained from the public website.
