# Supplementary material for: Association between intrinsic capacity and chronic lung disease among middle-aged and older adults: evidence from CHARLS and ELSA
Source: BMC Public Health. 2026 May 21;26:2211. doi: 10.1186/s12889-026-27756-9 (PMC13393715; doi:10.1186/s12889-026-27756-9)
Supplement: Supplementary file 1 — Supplementary Material 1. [file 12889_2026_27756_MOESM1_ESM.docx]

**Association Between Intrinsic Capacity and Incident Chronic Lung Disease Among Middle-aged and Older Adults: Evidence from CHARLS and ELSA**

**Content**

**Supplementary methods**

*Descriptions of covariates*

**Supplementary tables**

**Table S1.** The missing number and rate of covariates in baseline.

**Table S2.** Baseline of included and excluded participants.

**Table S3.** Baseline characteristics of study participant.

**Table S4.** Schoenfeld residuals test for the Cox proportional hazards assumption.

**Table S5.** Association of IC decline with risk of incident CLD with multiple imputation.

**Supplementary methods**

***Descriptions of covariates***

**Age**

Since the ELSA database mainly collects information on individuals over 50 years old, the CHARLS mainly includes the information of respondents aged 45 and above. In this study, to maintain consistency between the two datasets, we excluded individuals under the age of 50. In the study population, the age range of CHARLS participants was 50 to 101 years, whereas that of ELSA participants was 50 to 90 years. On this basis, we divided them according to whether their age was greater than 60 years old. They were classified into the > 60 years old group (code 1) and the ≤ 60 years old group (code 0).

**Marital status**

| **This study** | **CHARLS** | **ELSA** |
| --- | --- | --- |
| Married  (code 1) | 1.Married with spouse present; | 1.married or civil partner |
|  | 2.Married but not living with spouse temporarily for reasons such as work |  |
| Other (code 0) | 3.Separated | 4.separated， |
|  | 4.Divorced; | 5.divorced， |
|  | 5.Widowed | 7.widowed， |
|  | 6.Never married | 8.never married |

**Educational status**

| **This study** | **CHARLS** | **ELSA** |
| --- | --- | --- |
| High school and below (code 0) | 1.No formal education (illiterate) | 1.lt high-school |
|  | 2.Did not finish primary school but capable of reading and/or writing | 3.high-school graduate |
|  | 3.Sishu |  |
|  | 4.Elementary school |  |
|  | 5.Middle school |  |
|  | 6.High school |  |
| College and above  (code 1) | 7.Vocational school | 4.some college |
|  | 8.Two/Three Year College/Associate degree | 5.college and above |
|  | 9.Four Year College/Bachelor's degree |  |
|  | 11.Post-graduate (PhD) |  |

**Smoking status and Drinking status**

In CHARLS and ELSA, respondents will be asked whether they smoke or drink. In this study, individuals who answer "Yes" will be defined as those who smoke or drink (code 1), while those who answer "No" will be defined as those who do not (code 0).

**Sleeping time**

In CHARLS, respondents were asked about their average nightly sleep duration over the past month, whereas in ELSA, they were queried about their average nightly sleep duration over the past week. In this study, sleep duration was categorized into three groups: 6-9 hours (code 0), ≤6 hours (code 1), and ≥9 hours (code 2).

**Physical activity**

In CHARLS, physical activity encompasses light, moderate, and vigorous levels. Participants engaging in moderate or vigorous physical activity for more than 10 minutes per week were classified as one group (code 1), while the remaining participants were categorized as “Others” (code 0). In ELSA, individuals performing moderate or vigorous physical activity more than once per week were assigned to one group (code 1), with the rest defined as “Others” (code 0).

**Hypertension, Diabetes and Hearts disease**

Respondents were asked whether a physician had ever informed them of having specific conditions, such as high blood pressure or hypertension, diabetes, and heart disease. If the respondent answered “yes,” the condition was recorded as “1”; otherwise, it was recorded as “0”

**Body mass index**

The calculation of Body mass index (BMI) is based on measured height and weight. ELSA uses the World Health Organization (WHO) standards for classification (under 18.5 kg/m² (code 1), 18.5–25 kg/m² (code 0), 25–30 kg/m² (code 2), and greater than 30 kg/m² (code 3)), while CHARLS employs classification standards specific to the Chinese population (under 18.5 kg/m² (code 1), 18.5–24 kg/m² (code 0), 24–28 kg/m² (code 2), and greater than 28 kg/m² (code 3)).

**Table S1. The missing number and rate of covariates in baseline.**

| **Variable** | **CHARLS (n=3540)** | **ELSA (n=4357)** |
| --- | --- | --- |
| Age | 0(0.00%) | 0(0.00%) |
| BMI | 0(0.00%) | 0(0.00%) |
| CLD | 0(0.00%) | 0(0.00%) |
| Cancer | 6(0.17%) | 0(0.00%) |
| Diabetes | 18(0.51%) | 0(0.00%) |
| Drinking status | 199(5.62%) | 268(6.15%) |
| Education status | 0(0.00%) | 322(7.39%) |
| Heart disease | 10(0.28%) | 0(0.00%) |
| Hypertension | 14(0.40%) | 0(0.00%) |
| IC | 0(0.00%) | 0(0.00%) |
| Marital status | 0(0.00%) | 1(0.02%) |
| PA | 0(0.00%) | 0(0.00%) |
| Gender | 0(0.00%) | 0(0.00%) |
| Sleeping time | 7(0.20%) | 0(0.00%) |
| Smoking Status | 0(0.00%) | 0(0.00%) |

Abbreviations: CHARLS, China Health and Retirement Longitudinal Study; ELSA, English Longitudinal Study of Ageing, BMI, Body Mass Index; CLD, Chronic Lung Disease; PA, Physical Activity; IC, Intrinsic Capacity.

**Table S2. Baseline of included and excluded participants**

| **Variables** | **CHARLS** | | | | **Variables** | **ELSA** | | | |
| --- | --- | --- | --- | --- | --- | --- | --- | --- | --- |
|  | **Total**  **(n = 13391)** | **Exclude**  **(n = 9851)** | **Include**  **(n = 3540)** | ***P* value** |  | **Total**  **(n = 10372)** | **Exclude**  **(n = 6015)** | **Include**  **(n = 4357)** | ***P* value** |
| Age, n (%) |  |  |  | < 0.001 | Age, n (%) |  |  |  | 1 |
| ≤60 years | 6734 (50.288) | 4730 (48.015) | 2004 (56.61) |  | ≤60 years | 3126 (30.139) | 1813 (30.141) | 1313 (30.135) |  |
| > 60 years | 6657 (49.712) | 5121 (51.985) | 1536 (43.39) |  | > 60 years | 7246 (69.861) | 4202 (69.859) | 3044 (69.865) |  |
| Gender, n (%) |  |  |  | 0.004 | Gender, n (%) |  |  |  | 0.522 |
| Female | 6730 (50.258) | 5024 (51) | 1706 (48.192) |  | Female | 5693 (54.888) | 3285 (54.613) | 2408 (55.267) |  |
| Male | 6661 (49.742) | 4827 (49) | 1834 (51.808) |  | Male | 4679 (45.112) | 2730 (45.387) | 1949 (44.733) |  |
| Marital status, n (%) |  |  |  | < 0.001 | Marital status, n (%) |  |  |  | < 0.001 |
| Married | 11354 (84.801) | 8173 (82.983) | 3181 (89.859) |  | Married | 3416 (32.941) | 2149 (35.733) | 1267 (29.086) |  |
| Other | 2035 (15.199) | 1676 (17.017) | 359 (10.141) |  | Other | 6954 (67.059) | 3865 (64.267) | 3089 (70.914) |  |
| Education status, n (%) |  |  |  | 0.001 | Education status, n (%) |  |  |  | < 0.001 |
| High school and below | 13146 (98.2) | 9647 (97.969) | 3499 (98.842) |  | High school and below | 5381 (56.334) | 3391 (61.465) | 1990 (49.318) |  |
| College and above | 241 (1.8) | 200 (2.031) | 41 (1.158) |  | College and above | 4171 (43.666) | 2126 (38.535) | 2045 (50.682) |  |
| Smoking status, n (%) |  |  |  | 0.022 | Smoking status, n (%) |  |  |  | < 0.001 |
| No | 7811 (58.448) | 5800 (59.039) | 2011 (56.808) |  | No | 3860 (37.81) | 2062 (35.236) | 1798 (41.267) |  |
| Yes | 5553 (41.552) | 4024 (40.961) | 1529 (43.192) |  | Yes | 6349 (62.19) | 3790 (64.764) | 2559 (58.733) |  |
| Sleeping time, n (%) |  |  |  | < 0.001 | Sleeping time, n (%) |  |  |  | < 0.001 |
| 6 - 9 hours | 4824 (39.156) | 3322 (37.806) | 1502 (42.513) |  | 6 - 9 hours | 5896 (56.845) | 3122 (51.904) | 2774 (63.668) |  |
| ≤6 hours | 6473 (52.541) | 4711 (53.613) | 1762 (49.873) |  | ≤6 hours | 3874 (37.351) | 2512 (41.762) | 1362 (31.26) |  |
| ≥ 9 hours | 1023 (8.304) | 754 (8.581) | 269 (7.614) |  | ≥ 9 hours | 602 (5.804) | 381 (6.334) | 221 (5.072) |  |
| Drinking status, n (%) |  |  |  | < 0.001 | Drinking status, n (%) |  |  |  | < 0.001 |
| No | 9022 (72.466) | 6726 (73.839) | 2296 (68.722) |  | No | 1155 (13.322) | 782 (17.071) | 373 (9.122) |  |
| Yes | 3428 (27.534) | 2383 (26.161) | 1045 (31.278) |  | Yes | 7515 (86.678) | 3799 (82.929) | 3716 (90.878) |  |
| PA, n (%) |  |  |  | < 0.001 | PA, n (%) |  |  |  | < 0.001 |
| Moderate-to-vigorous | 10123 (75.596) | 7640 (77.556) | 2483 (70.141) |  | Moderate-to-vigorous | 3912 (37.717) | 2800 (46.55) | 1112 (25.522) |  |
| Other | 3268 (24.404) | 2211 (22.444) | 1057 (29.859) |  | Other | 6460 (62.283) | 3215 (53.45) | 3245 (74.478) |  |
| BMI, n (%) |  |  |  | < 0.001 | BMI, n (%) |  |  |  | < 0.001 |
| 18.5-24 kg/m2 | 5692 (53.973) | 3838 (54.782) | 1854 (52.373) |  | 18.5-25 kg/m2 | 2036 (26.569) | 875 (26.467) | 1161 (26.647) |  |
| <18.5 kg/m2 | 848 (8.041) | 672 (9.592) | 176 (4.972) |  | <18.5 kg/m2 | 69 (0.9) | 39 (1.18) | 30 (0.689) |  |
| 24.0 - 28.0 kg/m2 | 2900 (27.499) | 1792 (25.578) | 1108 (31.299) |  | 25.0 - 30.0 kg/m2 | 3160 (41.237) | 1261 (38.143) | 1899 (43.585) |  |
| >28.0 kg/m2 | 1106 (10.487) | 704 (10.049) | 402 (11.356) |  | >30.0 kg/m2 | 2398 (31.293) | 1131 (34.211) | 1267 (29.08) |  |
| Diabetes, n (%) |  |  |  | 0.646 | Diabetes, n (%) |  |  |  | < 0.001 |
| No | 12343 (93.113) | 9070 (93.179) | 3273 (92.93) |  | No | 9209 (88.796) | 5207 (86.581) | 4002 (91.852) |  |
| Yes | 913 (6.887) | 664 (6.821) | 249 (7.07) |  | Yes | 1162 (11.204) | 807 (13.419) | 355 (8.148) |  |
| Hypertension, n (%) |  |  |  | 0.003 | Hypertension, n (%) |  |  |  | < 0.001 |
| No | 9447 (70.955) | 6876 (70.249) | 2571 (72.915) |  | No | 5969 (57.555) | 3257 (54.157) | 2712 (62.245) |  |
| Yes | 3867 (29.045) | 2912 (29.751) | 955 (27.085) |  | Yes | 4402 (42.445) | 2757 (45.843) | 1645 (37.755) |  |
| Heart disease, n (%) |  |  |  | 0.004 | Heart disease, n (%) |  |  |  | < 0.001 |
| No | 11466 (86.243) | 8370 (85.714) | 3096 (87.705) |  | No | 8361 (80.619) | 4647 (77.27) | 3714 (85.242) |  |
| Yes | 1829 (13.757) | 1395 (14.286) | 434 (12.295) |  | Yes | 2010 (19.381) | 1367 (22.73) | 643 (14.758) |  |
| Cancer, n (%) |  |  |  | 0.123 | Cancer, n (%) |  |  |  | < 0.001 |
| No | 13179 (99.001) | 9672 (98.916) | 3507 (99.236) |  | No | 9276 (89.442) | 5327 (88.577) | 3949 (90.636) |  |
| Yes | 133 (0.999) | 106 (1.084) | 27 (0.764) |  | Yes | 1095 (10.558) | 687 (11.423) | 408 (9.364) |  |
| CLD, n (%) |  |  |  | < 0.001 | CLD, n (%) |  |  |  | < 0.001 |
| No | 10468 (78.172) | 7375 (74.865) | 3093 (87.373) |  | No | 6251 (92.143) | 2028 (83.56) | 4223 (96.924) |  |
| Yes | 2923 (21.828) | 2476 (25.135) | 447 (12.627) |  | Yes | 533 (7.857) | 399 (16.44) | 134 (3.076) |  |
| IC, n (%) |  |  |  | 0.024 | IC, n (%) |  |  |  | < 0.001 |
| Normal | 1828 (34.111) | 583 (32.051) | 1245 (35.169) |  | Normal | 4237 (65.205) | 1200 (56.049) | 3037 (69.704) |  |
| Impaired | 3531 (65.889) | 1236 (67.949) | 2295 (64.831) |  | Impaired | 2261 (34.795) | 941 (43.951) | 1320 (30.296) |  |

***Abbreviations***: CHARLS, China Health and Retirement Longitudinal Study; ELSA, English Longitudinal Study of Ageing, BMI, Body Mass Index; CLD, Chronic Lung Disease; PA, Physical Activity; IC, Intrinsic Capacity.

**Table S3. Baseline characteristics of study participants.**

| **Variables** | **CHARLS** | | | | **Variables** | **ELSA** | | | |
| --- | --- | --- | --- | --- | --- | --- | --- | --- | --- |
|  | **Total**  **(n = 3540)** | **IC normal**  **(n = 1245)** | **IC impaired**  **(n = 2295)** | ***P* value** |  | **Total**  **(n = 4357)** | **IC normal**  **(n = 3037)** | **IC impaired**  **(n = 1320)** | ***P* value** |
| Age, n (%) |  |  |  | < 0.001 | Age, n (%) |  |  |  | < 0.001 |
| ≤60 years | 2004 (56.61) | 753 (60.48) | 1251 (54.51) |  | ≤60 years | 1313 (30.14) | 968 (31.87) | 345 (26.14) |  |
| > 60 years | 1536 (43.39) | 492 (39.52) | 1044 (45.49) |  | > 60 years | 3044 (69.86) | 2069 (68.13) | 975 (73.86) |  |
| Gender, n (%) |  |  |  | < 0.001 | Gender, n (%) |  |  |  | 0.029 |
| Female | 1706 (48.19) | 461 (37.03) | 1245 (54.25) |  | Female | 2408 (55.27) | 1645 (54.17) | 763 (57.8) |  |
| Male | 1834 (51.81) | 784 (62.97) | 1050 (45.75) |  | Male | 1949 (44.73) | 1392 (45.83) | 557 (42.2) |  |
| Marital status, n (%) |  |  |  | < 0.001 | Marital status, n (%) |  |  |  | < 0.001 |
| Married | 3181 (89.86) | 1160 (93.17) | 2021 (88.06) |  | Married | 1267 (29.09) | 758 (24.97) | 509 (38.56) |  |
| Other | 359 (10.14) | 85 (6.83) | 274 (11.94) |  | Other | 3089 (70.91) | 2278 (75.03) | 811 (61.44) |  |
| Education status, n (%) |  |  |  | 0.003 | Education status, n (%) |  |  |  | < 0.001 |
| High school and below | 3499 (98.84) | 1221 (98.07) | 2278 (99.26) |  | High school and below | 1990 (49.32) | 1269 (44.97) | 721 (59.44) |  |
| College and above | 41 (1.16) | 24 (1.93) | 17 (0.74) |  | College and above | 2045 (50.68) | 1553 (55.03) | 492 (40.56) |  |
| Smoking status, n (%) |  |  |  | < 0.001 | Smoking status, n (%) |  |  |  | 0.079 |
| No | 2011 (56.81) | 636 (51.08) | 1375 (59.91) |  | No | 1798 (41.27) | 1280 (42.15) | 518 (39.24) |  |
| Yes | 1529 (43.19) | 609 (48.92) | 920 (40.09) |  | Yes | 2559 (58.73) | 1757 (57.85) | 802 (60.76) |  |
| Sleeping time, n (%) |  |  |  | < 0.001 | Sleeping time, n (%) |  |  |  | < 0.001 |
| 6 - 9 hours | 1502 (42.51) | 626 (50.32) | 876 (38.27) |  | 6 - 9 hours | 2774 (63.67) | 2067 (68.06) | 707 (53.56) |  |
| ≤6 hours | 1762 (49.87) | 533 (42.85) | 1229 (53.69) |  | ≤6 hours | 1362 (31.26) | 825 (27.16) | 537 (40.68) |  |
| ≥ 9 hours | 269 (7.61) | 85 (6.83) | 184 (8.04) |  | ≥ 9 hours | 221 (5.07) | 145 (4.77) | 76 (5.76) |  |
| Drinking status, n (%) |  |  |  | < 0.001 | Drinking status, n (%) |  |  |  | < 0.001 |
| No | 2296 (68.72) | 729 (62.95) | 1567 (71.78) |  | No | 373 (9.12) | 201 (7) | 172 (14.12) |  |
| Yes | 1045 (31.28) | 429 (37.05) | 616 (28.22) |  | Yes | 3716 (90.88) | 2670 (93) | 1046 (85.88) |  |
| PA, n (%) |  |  |  | 1 | PA, n (%) |  |  |  | < 0.001 |
| Moderate-to-vigorous | 2483 (70.14) | 873 (70.12) | 1610 (70.15) |  | Moderate-to-vigorous | 1112 (25.52) | 651 (21.44) | 461 (34.92) |  |
| Other | 1057 (29.86) | 372 (29.88) | 685 (29.85) |  | Other | 3245 (74.48) | 2386 (78.56) | 859 (65.08) |  |
| BMI, n (%) |  |  |  | < 0.001 | BMI, n (%) |  |  |  | < 0.001 |
| 18.5-24 kg/m2 | 1854 (52.37) | 661 (53.09) | 1193 (51.98) |  | 18.5-25 kg/m2 | 1161 (26.65) | 853 (28.09) | 308 (23.33) |  |
| <18.5 kg/m2 | 176 (4.97) | 0 (0) | 176 (7.67) |  | <18.5 kg/m2 | 30 (0.69) | 0 (0) | 30 (2.27) |  |
| 24.0 - 28.0 kg/m2 | 1108 (31.3) | 423 (33.98) | 685 (29.85) |  | 25.0 - 30.0 kg/m2 | 1899 (43.59) | 1347 (44.35) | 552 (41.82) |  |
| >28.0 kg/m2 | 402 (11.36) | 161 (12.93) | 241 (10.5) |  | >30.0 kg/m2 | 1267 (29.08) | 837 (27.56) | 430 (32.58) |  |
| Diabetes, n (%) |  |  |  | 0.165 | Diabetes, n (%) |  |  |  | < 0.001 |
| No | 3273 (92.93) | 1162 (93.79) | 2111 (92.47) |  | No | 4002 (91.85) | 2827 (93.09) | 1175 (89.02) |  |
| Yes | 249 (7.07) | 77 (6.21) | 172 (7.53) |  | Yes | 355 (8.15) | 210 (6.91) | 145 (10.98) |  |
| Hypertension, n (%) |  |  |  | 0.156 | Hypertension, n (%) |  |  |  | < 0.001 |
| No | 2571 (72.92) | 924 (74.4) | 1647 (72.11) |  | No | 2712 (62.24) | 1980 (65.2) | 732 (55.45) |  |
| Yes | 955 (27.08) | 318 (25.6) | 637 (27.89) |  | Yes | 1645 (37.76) | 1057 (34.8) | 588 (44.55) |  |
| Heart disease, n (%) |  |  |  | 0.009 | Heart disease, n (%) |  |  |  | < 0.001 |
| No | 3096 (87.71) | 1116 (89.71) | 1980 (86.61) |  | No | 3714 (85.24) | 2645 (87.09) | 1069 (80.98) |  |
| Yes | 434 (12.29) | 128 (10.29) | 306 (13.39) |  | Yes | 643 (14.76) | 392 (12.91) | 251 (19.02) |  |
| Cancer, n (%) |  |  |  | 0.996 | Cancer, n (%) |  |  |  | 0.033 |
| No | 3507 (99.24) | 1232 (99.19) | 2275 (99.26) |  | No | 3949 (90.64) | 2772 (91.27) | 1177 (89.17) |  |
| Yes | 27 (0.76) | 10 (0.81) | 17 (0.74) |  | Yes | 408 (9.36) | 265 (8.73) | 143 (10.83) |  |
| CLD, n (%) |  |  |  | < 0.001 | CLD, n (%) |  |  |  | < 0.001 |
| No | 3093 (87.37) | 1143 (91.81) | 1950 (84.97) |  | No | 4223 (96.92) | 2969 (97.76) | 1254 (95) |  |
| Yes | 447 (12.63) | 102 (8.19) | 345 (15.03) |  | Yes | 134 (3.08) | 68 (2.24) | 66 (5) |  |

***Abbreviations***: CHARLS, China Health and Retirement Longitudinal Study; ELSA, English Longitudinal Study of Ageing, BMI, Body Mass Index; CLD, Chronic Lung Disease; PA, Physical Activity; IC, Intrinsic Capacity.

**Table S4. Schoenfeld residuals test for the Cox proportional hazards assumption**

| Variables | CHARLS | | | ELSA | | |
| --- | --- | --- | --- | --- | --- | --- |
|  | **χ²** | **df** | **P value** | **χ²** | **df** | **P value** |
| IC | 1.238 | 1 | 0.266 | 2.399 | 1 | 0.121 |
| Age | 1.500 | 1 | 0.221 | 1.611 | 1 | 0.204 |
| Gender | 2.477 | 1 | 0.116 | 5.606 | 1 | 0.018 |
| Education status | 2.718 | 1 | 0.099 | 1.153 | 1 | 0.283 |
| Marital status | 3.992 | 1 | 0.046 | 3.343 | 1 | 0.067 |
| Smoking status | 5.076 | 1 | 0.024 | 0.016 | 1 | 0.899 |
| Drinking status | 0.141 | 1 | 0.708 | 0.600 | 1 | 0.438 |
| Sleeping time | 0.329 | 1 | 0.566 | 0.857 | 1 | 0.355 |
| PA | 0.167 | 1 | 0.683 | 1.499 | 1 | 0.221 |
| Hypertension | 0.337 | 1 | 0.561 | 0.311 | 1 | 0.577 |
| Diabetes | 2.279 | 1 | 0.131 | 2.103 | 1 | 0.147 |
| BMI | 0.227 | 1 | 0.634 | 0.478 | 1 | 0.489 |
| Heart disease | 1.629 | 1 | 0.202 | 1.235 | 1 | 0.266 |
| Cancer | 1.667 | 1 | 0.197 | 0.084 | 1 | 0.772 |
| GLOBAL | 23.218 | 14 | 0.057 | 16.056 | 14 | 0.310 |

***Abbreviations***: CHARLS, China Health and Retirement Longitudinal Study; ELSA, English Longitudinal Study of Ageing, BMI, Body Mass Index; CLD, Chronic Lung Disease; PA, Physical Activity; IC, Intrinsic Capacity.

**Table S5. Association of IC impaired with risk of incident CLD with multiple imputation.**

| **Term** | **Event/n** | **Model 1** | | **Model 2** | | **Model 3** | | **Model 4** | |
| --- | --- | --- | --- | --- | --- | --- | --- | --- | --- |
|  |  | **HR (95%CI)** | ***P* value** | **HR (95%CI)** | ***P* value** | **HR (95%CI)** | ***P* value** | **HR (95%CI)** | ***P* value** |
| **CHARLS** |  |  |  |  |  |  |  |  |  |
| IC normal | 102/1245 | Ref |  | Ref |  | Ref |  | Ref |  |
| IC impaired | 345/2295 | 1.902(1.524-2.374) | <0.001 | 1.942(1.551-2.431) | <0.001 | 1.921(1.533-2.406) | <0.001 | 1.894(1.512-2.373) | <0.001 |
| **ELSA** |  |  |  |  |  |  |  |  |  |
| IC normal | 68/3037 | Ref |  | Ref |  | Ref |  | Ref |  |
| IC impaired | 66/1320 | 2.275(1.617-3.203) | <0.001 | 2.119(1.495-3.004) | <0.001 | 1.943(1.362-2.773) | <0.001 | 1.913(1.338-2.736) | <0.001 |

**Abbreviations:** CHARLS, China Health and Retirement Longitudinal Study; CI, Confidence Interval; ELSA, English Longitudinal Study of Ageing; HR, Hazard Ratio; IC, Intrinsic Capacity; Ref, Reference.

Model 1: unadjusted.

Model 2: adjusted for Age, Gender, Education status and Marital status.

Model 3: adjusted for Age, Gender, Education status, Marital status, Smoking status, Drinking status, Sleeping time, and Physical Activity

Model 4: adjusted for Age, Gender, Education status, Marital status, Smoking status, Drinking status, Sleeping time, Physical activity, Hypertension, Diabetes, BMI, Heart disease and Cancer.
